# A whole-genome assembly of the domestic cow, *Bos taurus*

**DOI:** 10.1186/gb-2009-10-4-r42

**Published:** 2009-04-24

**Authors:** Aleksey V Zimin, Arthur L Delcher, Liliana Florea, David R Kelley, Michael C Schatz, Daniela Puiu, Finnian Hanrahan, Geo Pertea, Curtis P Van Tassell, Tad S Sonstegard, Guillaume Marçais, Michael Roberts, Poorani Subramanian, James A Yorke, Steven L Salzberg

**Affiliations:** 1 Institute for Physical Science and Technology, University of Maryland, College Park, Maryland 20742, USA; 2 Center for Bioinformatics and Computational Biology, University of Maryland, College Park, Maryland 20742, USA; 3 Agricultural Research Service, U.S. Department of Agriculture, 10300 Baltimore Ave., Beltsville, Maryland 20705, USA

## Abstract

**Background:**

The genome of the domestic cow, *Bos taurus*, was sequenced using a mixture of hierarchical and whole-genome shotgun sequencing methods.

**Results:**

We have assembled the 35 million sequence reads and applied a variety of assembly improvement techniques, creating an assembly of 2.86 billion base pairs that has multiple improvements over previous assemblies: it is more complete, covering more of the genome; thousands of gaps have been closed; many erroneous inversions, deletions, and translocations have been corrected; and thousands of single-nucleotide errors have been corrected. Our evaluation using independent metrics demonstrates that the resulting assembly is substantially more accurate and complete than alternative versions.

**Conclusions:**

By using independent mapping data and conserved synteny between the cow and human genomes, we were able to construct an assembly with excellent large-scale contiguity in which a large majority (approximately 91%) of the genome has been placed onto the 30 *B. taurus *chromosomes. We constructed a new cow-human synteny map that expands upon previous maps. We also identified for the first time a portion of the *B. taurus *Y chromosome.

## Abstract 2

A cow whole-genome assembly of 2.86 billion base pairs that closes gaps and corrects previously-described inversions and deletions as well as describing a portion of the Y chromosome.

## Background

Seven years after the first whole-genome assembly of the human genome [1], sequencing and assembly of mammalian genomes has become almost routine. However, despite the continuing progress on sequencing technology, the assembly problem is far from solved. Assemblies of large genomes contain numerous errors, and many years of work can be dedicated to correcting errors and improving an assembly [2]. Technical progress in computational assembly methods offers the potential to make many of these improvements far faster and more efficiently than would be possible by laboratory methods.

Having an accurate assembly of the genome of an important species provides an invaluable substrate for future research. For example, studies of genetic diversity need a good reference genome in order to catalog differences in new strains or lineages. Expression analyses that sequence RNA from various tissues rely on the genome to map out gene models and to discover such features as alternative splicing. Creating a more complete, accurate reference genome avoids much wasted effort that might result from attempts to use erroneous polymorphisms or other errors. For these reasons, the human genome program expended substantial efforts to improve the original human 'draft' assembly, which had 147,821 gaps and was missing 10% of the euchromatic regions, to a 'near-complete' draft three years later, with just 341 gaps and less than 1% of the euchromatin still missing [3]. As that study pointed out, an improved assembly "greatly improves the precision of biological analyses ... including studies of gene number, birth and death."

To assemble the genome of the domestic cow, *Bos taurus*, we have augmented the latest assembly software with additional post-processing algorithms that utilize paired-end sequence information, mapping data, and synteny with the human genome to detect errors, correct inverted segments, and fill gaps in the sequence. With the help of extensive marker data, we were able to anchor approximately 91% of the assembled genome onto chromosomes. The resulting assembly provides a very high-quality resource for annotation and ongoing studies in the genetics of the domestic cow as well as comparative mammalian genomics.

## Results and discussion

**Figure 1:**
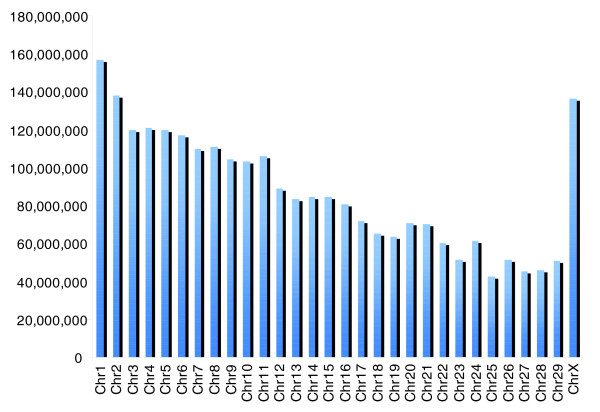
Chromosome (Chr) lengths (in base pairs) based on amount of sequence in the *B. taurus *assembly placed on each chromosome.

Our assembly of the *B. taurus *genome contains 2,857,605,192 bp, of which 2,612,820,649 bp are placed on one of the 30 chromosomes (Table 1). The remaining 245 Mbp are contained in unplaced contiguous sequences (contigs). Figure 1 shows the amount of sequence placed in each of the 29 autosomes and chromosome X. As the figure shows, length is inversely correlated with chromosome number, with a few exceptions, including chromosomes 11, 20, and 24.

**Table 1:** Overall assembly statistics for the UMD2 assembly of *B. taurus*

Total size of all contigs	2,857,605,192
Total size of all placed contigs	2,612,820,649
Total size of unplaced contigs	244,784,543
N50 contig size (based on 2.5 Gb genome size)	93,156
N50 contig count	7,906
Number of contigs >10,000 bp	44,433
Total size of contigs >10,000 bp	2,563,627,935

N50 contig size is the value X such that at least half of the genome is contained in contigs of size X or larger. N50 contig count is the number of contigs of size X or larger.

**Figure 2:**
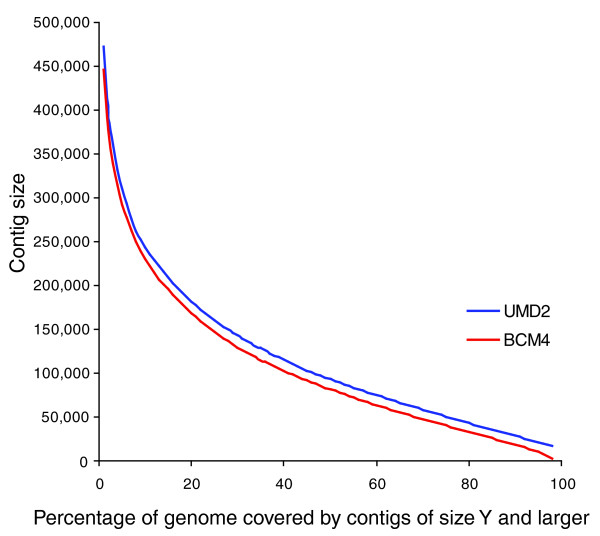
Cumulative plot of the N statistic for both the UMD2 (blue) and BCM4 (red) assemblies. Each point (X, Y) in the plot shows the contig size Y such that X% of the genome is contained in contigs of length Y or larger, for a genome of size 2.5 Gbp. For example, the N50 size for each assembly corresponds to the value of Y at X = 50; for UMD2 this value is 93,156 and for BCM4 it is 81,627.

We evaluated our assembly (University of Maryland assembly of *B. taurus*, release 2 (UMD2)) for completeness and correctness in several ways, comparing it to independent mapping data, to independently sequenced mRNA data, and to the alternative draft assembly produced by the Baylor College of Medicine Human Genome Sequencing Center, BosTau4.0 (BCM4). Each of the assemblies contains both 'placed' sequence, for which the location on the chromosomes is known, and 'unplaced' sequence. As shown in Table 2, the UMD2 assembly is larger than BCM4, with approximately 150 Mb (6%) more sequence placed onto chromosomes. In addition to total size, the N50 size is a very useful statistic for comparing genome assemblies: it represents the size *N *such that 50% of the genome is contained in contigs of size *N *or greater. For UMD2, the N50 contig size is 93,156 bp, while for BCM4 the N50 size is 81,627, approximately 14% smaller. Figure 2 shows that for all values from N1 to N98, the UMD2 assembly is larger than BCM4.

**Table 2:** Comparison of the *B. taurus *UMD2 and BCM4 assemblies according to sequence and mapping statistics

Assembly	UMD2	BCM4
Total sequence placed on chromosomes (Gbp)	2.61	2.47
N50 contig size (bp)	93,156	81,627
N50 contig count	7,906	8,712
Total Cmap markers mapped to placed sequence	14,620	13,699
Cmap markers mapping to the wrong chromosome	119	82

N50 statistics are based on a genome size of 2.5 Gbp.

One of the most striking differences between the BCM4 and UMD2 assemblies is the assembly of the *B. taurus *X chromosome (BtX). UMD2 assigned 136 Mbp of sequence to the X chromosome, while the BCM4 assembly assigned only 83 Mbp. As we describe below, all sequence on BtX in our assembly is homologous to the human X chromosome (HsX).

Independently generated mapping data provide another measure of the quality of the assembly. Snelling *et al*. [4] created a *B. taurus *map from three radiation hybrid panels, two genetic maps, and bacterial artificial chromosome (BAC) end sequences. We aligned all of the 17,254 markers (of which 17,193 are unique) in their composite map (Cmap) to both assemblies. A marker was considered as matching a chromosome if 90% of the marker sequence aligned with at least 95% identity. Of the Cmap markers, 14,620 align to the UMD2 assembly's chromosomes, versus 13,699 markers (6.3% fewer) for the BCM4 assembly. A small number of Cmap markers (119 and 82 for UMD2 and BCM4, respectively) mapped to a different chromosome from the one indicated in the Cmap data.

One likely reason for the larger size and greater genome coverage of our assembly is the BAC-based assembly strategy employed by the Atlas assembler used to build BCM4 [5]. That strategy involved breaking the genome into BAC-sized pieces, assembling those pieces using BAC reads and whole-genome shotgun (WGS) reads, and then merging the results. This strategy fails to incorporate reads that fall outside the regions covered by BACs. We estimate that at least 2% of the UMD2 assembly is missing from BCM4 due to gaps between BACs.

**Figure 3:**
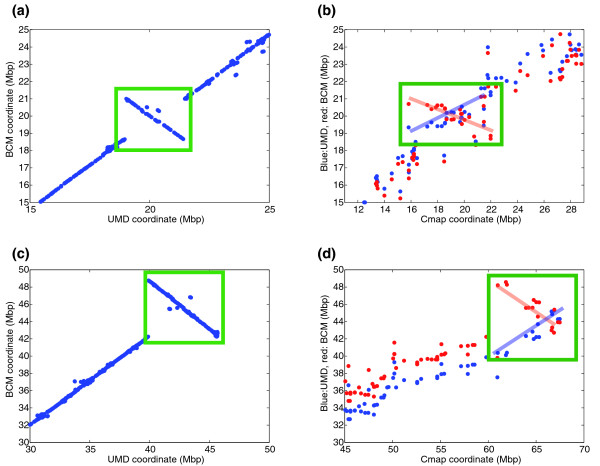
Examples of large-scale disagreements between UMD2 and BCM4. **(a) **Dot-plot alignment of the region between 15 Mbp and 25 Mbp of chromosome 26 showing a large inversion in BCM4 compared to UMD2; **(b) **positions of Cmap markers for the same region of chromosome 26, plotted against their positions in UMD2 (blue) and BCM4 (red), showing that Cmap supports the UMD2 assembly. **(c) **Alignment of 7 Mbp of chromosome 27, showing a large inversion in BCM4 compared to UMD2; **(d) **positions of Cmap markers for the same region of chromosome 27, showing as in (b) that Cmap is in much closer agreement with the UMD2 assembly.

We directly aligned the two assemblies against each other in order to detect any major disagreements. Ten of the 30 chromosomes contain one or more large (>500 kb) discrepancies, primarily inversions but also deletions and translocations. Figure 3 illustrates two relatively large inversions, spanning 4 and 2.5 Mbp, on chromosomes 26 and 27. In both of these cases, as in all other large discrepancies, the Cmap data support the UMD2 assembly. Alignment plots for all 30 chromosomes are provided online in Additional data file 2.

We conducted a comparison between the two assemblies for differences in the number of apparent segmental duplications, focusing on the types of duplications that might confound assembly. We collected all intra-chromosomal duplicated segments from both assemblies that were >5 kb in length and >95% identical. We found that UMD2 had significantly fewer duplications of this type, 662 versus 3,098 in BCM4. If these regions were incorrectly collapsed duplications in UMD2, then coverage by WGS reads should be higher (approximately twice the genome-wide level) and mate pairs flanking the regions would show inconsistencies [6]. However, after analyzing regions that are single-copy in UMD2 and duplicated in BCM4, we found no substantial discrepancies in either mate pairs or coverage, indicating that the regions are most likely single-copy. It is possible that BCM4 failed to merge overlapping BACs (from different haplotypes), which would give the appearance of segmental duplications; further analysis will be necessary to resolve this question.

**Figure 4:**
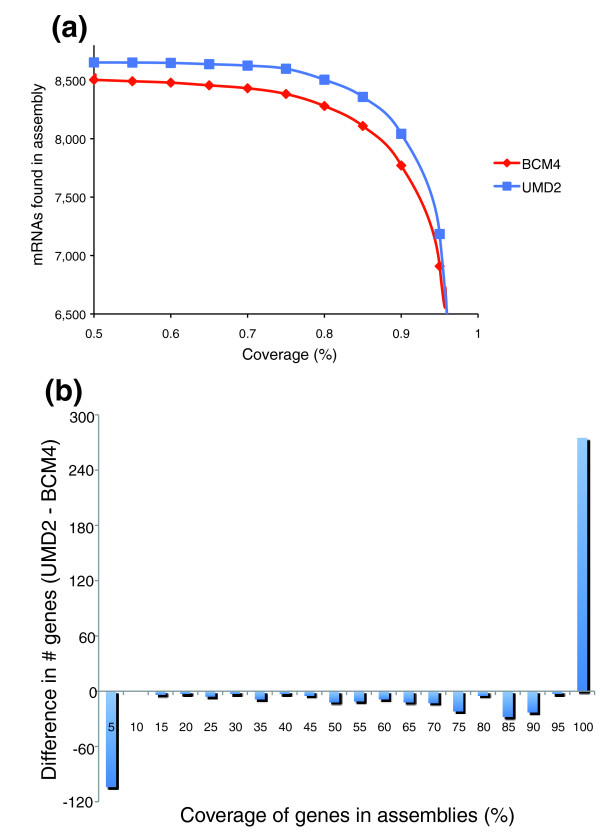
Assembly comparison by gene mapping. **(a) **Number of RefSeq mRNA sequences (out of 8,689) that can be aligned to each genome assembly at varying coverage cutoffs (horizontal axis) with at least 95% sequence identity. **(b) **Difference in the number of mRNAs mapping to the two assemblies at different levels of coverage, plotted as UMD2 minus BCM4. Negative values indicate that BCM4 has more genes at a given level, while positive values indicate that UMD2 has more. For example, at 0-5% coverage, 104 more mRNAs map to BCM4 than to UMD2. At 95-100% coverage, 275 more mRNAs map to UMD2. Blue, UMD2 assembly; red, BCM4 assembly.

Another indicator of assembly completeness, and also of its potential for annotation, is the extent to which known gene sequences can be mapped onto it. We aligned 8,689 independently validated full-length cow mRNA sequences to the two assemblies, using spliced alignment mapping tools (see Materials and methods). Figure 4a and Table S1 in Additional data file 1 show the number of sequences that had more than a fraction *f *of their bases contained in each genome for a range of *f *values. When all alignments of a gene are considered, UMD2 contains at least a portion of 8,659 mRNAs, compared to 8,555 for BCM4. All but two of the genes that map to BCM4 can be found in UMD2, whereas 106 are unique to UMD2 and not found in BCM4. Together, the two assemblies contain all but 28 of the mRNA sequences, as well as paralogs of 25 of the remaining 28 genes. More significant differences between the two genomes become apparent when the aligned fraction of the gene is considered. For instance, 8,042 genes have more than 90% of their bases mapped to the UMD2 genome, compared to only 7,771 genes for BCM4. We also directly compared the distributions of gene coverage between the two assemblies, shown in Figure 4b. BCM4 has relatively more genes with low coverage, while UMD2 has a greater number of genes at the highest level (95-100%) of coverage. Overall, UMD2 has a more complete representation of the genes while containing nearly every gene in BCM4, and therefore provides a more comprehensive resource for gene annotation.

### Single nucleotide differences

In a base-by-base comparison, the UMD2 and BCM4 assemblies have >2.0 million single-nucleotide differences (SNDs). Some of these might be valid haplotype differences, in which the two assemblies are both correct, while others might be errors. We focused our analysis on a subset of positions where the underlying read data indicated that the position was highly likely to be homozygous, because a large majority (or all) reads agreed with one another. We also required that each SND was flanked by 50-bp exact matches in both assemblies (see Materials and methods), which reduced the set of SNDs to 389,015. We then looked for cases where no more than one read confirmed one assembly, and all other reads (at least three) confirmed the other assembly. The UMD2 assembly contains 10,636 instances of these apparent errors versus 30,750 in the BCM4 assembly. Thus, there were approximately three times more apparently erroneous SNDs in the BCM4 assembly.

Another way to look at fine-grain accuracy is to compare the assembly to independently generated sequences. We compared both assemblies to six finished BACS, from a different cow than the source of the whole-genome project. These BAC clones were not used in either the UMD2 or BCM4 assemblies. Ninety-six percent of the BAC sequence is contained in UMD2, versus 91% in BCM4. Considering only the portions of the BAC sequence that matched, the average disagreement between the BACs and UMD2 was 0.58%, whereas for BCM4 the discrepancy rate was 0.96%. Although some of these mismatches are likely due to true polymorphisms, the excess discrepancies in BCM4 are likely to represent erroneous base calls, indicating a higher error rate in BCM4.

### The *B. taurus *Y chromosome

Because two-thirds of the data came from a female cow, and the male DNA was based on a BAC library (Materials and methods), only a very limited amount of the assembly can be assigned to the Y chromosome. (It is worth noting here that the BCM4 assembly does not assign any sequence to the Y chromosome.) We aligned all unplaced contigs to the human Y chromosome in an effort to identify *B. taurus *Y sequence, and we identified 71 contigs that map to Y. When contigs in the same scaffolds were included, the total increased to 94 contigs, covering 832,527 bp. These contigs include a portion of the male sex determination gene *SRY *[7]. Because few of these contigs are currently ordered with respect to one another, further work will be required to construct a better picture of the Y chromosome's structure.

### Comparison to the human genome

Although humans are closer to mice than to cows, cows and humans have sufficient DNA sequence similarity to enable us to map the human genome almost entirely onto cow. Previous efforts based on mapping data showed that human and cow have approximately 201 homologous blocks of DNA [8]. We used flexible criteria (see Materials and methods) to align all cow chromosomes to all human chromosomes, creating a new, high-resolution synteny map of human and cow. A region was considered a homologous synteny block (HSB) if the human-cow alignment extended for at least 250 Kbp and if it was not interrupted by an inversion or by an HSB on another chromosome. If two HSBs were interrupted by a gap of <3 Mbp and nothing else fell in that gap, the two blocks were merged. (Note that if a large region of synteny is interrupted by a distinct HSB, the interruption creates three HSBs.) A modified Oxford grid, shown in Table 3, shows the numbers of syntenic blocks shared between all human and cow chromosomes.

**Table 3:** Modified Oxford grid showing the number of homologous synteny blocks on each chromosome of the cow (*B. taurus*) and human genomes

	**Human chromosome**																						
	
**Cow chromosome**	1	2	3	4	5	6	7	8	9	10	11	12	13	14	15	16	17	18	19	20	21	22	X
1			**4**																		**4**		
2	**1**	**4**							**1**														
3	**5**	**1**					**1**																
4							**12**																
5												**6**										**5**	
6				**5**																			
7					**5**														**5**				
8				**1**				**5**	**10**														
9						**3**																	
10					**4**									**5**	**5**								
11		**8**							**1**														
12													**5**										
13										**7**										**7**			
14								**3**															
15											**6**												
16	**10**																						
17				**3**								**5**										**4**	
18																**4**			**4**				
19																	**20**						
20					**2**																		
21														**2**	**17**								
22			**8**																				
23						**3**																	
24																		**4**					
25							**7**									**7**							
26										**3**													
27			**1**	**1**				**4**															
28	**3**									**6**													
29											**7**												
X																							**14**

**Figure 5:**
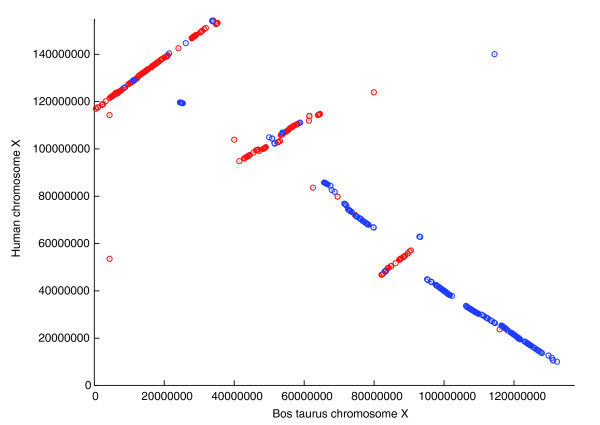
Aligment of *B. taurus *chromosome X to human chromosome X, showing regions of large-scale synteny. Most of the two chromosomes is shared in the five large blocks evident in the figure. Red: sequences are aligned in the same orientation; blue: sequences are aligned, but one is in the reverse complement orientation. The inverted (blue) block at approximately 25 Mbp in *B. taurus*, although small at this scale, spans over 800 Kbp.

Our new, more-detailed map largely agrees with previously identified blocks, with a number of important differences. In a few cases, our map has fewer HSBs between a pair of chromosomes, but in many more cases, we detected new synteny blocks that had been missed previously; most of these were inversions or interruptions in larger blocks. Overall, our map increases the total number of HSBs to 268. These were created from 245 evolutionary breakpoints (268 minus 23 human chromosomes) that have appeared since the divergence of human and cow. For example, BtX and HsX were previously reported to share seven HSBs [8]. Figure 5, which shows the alignment of BtX and HsX, reveals that five large blocks cover most of the two chromosomes, with one additional, much smaller block of 800 Kbp spanning the region from approximately 24.5 Mbp to 25.3 Mbp in BtX. Not visible on this scale, though, are seven additional inversions, bringing the total number of HSBs for the X chromosome to 14. We found no HSBs on BtX that mapped to any other human chromosomes besides X.

We also considered how many human genes can be found in the cow genome. For this analysis, we only considered curated human genes from the National Center for Biotechnology Information (NCBI) RefSeq database. We identified 25,710 RefSeq proteins representing 18,019 distinct human genes (many with alternative isoforms), and aligned these to the cow genome. Of the 18,019 human genes, 17,253 (95.7%) mapped to cow using our criteria. This left 766 genes that failed to map. Of these, 111 are annotated as 'hypothetical' proteins and may represent inaccurate gene models in human. The remaining 655 human genes failed to map either because they are too divergent or because the cow assembly is too fragmented or contains gaps in the regions containing those genes. Using the identical methods, we found that 17,107 human genes mapped onto the BCM4 assembly. Of the unmapped genes, 693 failed to map onto either assembly, 219 mapped onto UMD2 but not on BCM4, and 73 mapped onto BCM4 but not UMD2.

One surprising result was our finding that the initial assembly contained two unusual contaminants, *Acinetobacter baumannii *and *Serratia marcescens*. These bacteria are not used as sequencing reagents and are not usually detected when screening for contaminants; they appear to represent environmental contamination. The bacterial contigs, totaling 43,311 bp in 14 contigs, were removed from the UMD2 assembly, but are provided on our ftp site [9].

## Conclusions

These results illustrate how the information contained in the read data for a whole-genome sequencing project provide a valuable resource for continuing improvements to a genome, and how independently generated data can be merged into WGS data to produce a better assembly. The resulting improvements should provide immediate benefits to the research community, with whom we hope to work to improve the assembly further. Until the assembly is truly finished - a state that no mammalian genome, including human, has yet reached - we will continue to incorporate new data to fill in gaps, to correct mis-oriented regions, and to place more sequence onto chromosomes. The genomes of alpaca and sheep, which are currently being sequenced, should provide a rich source for making further improvements based on evolutionary conservation between these closely related mammals.

## Materials and methods

### Initial assembly

We downloaded approximately 37 million *B. taurus *reads from the NCBI Trace Archive. The original sequencing was conducted at the Baylor College of Medicine, and the BCM4 assembly was produced by the Atlas assembly program [5] and released to the public in October 2007. BCM4 was the fourth and final assembly, with previous releases occurring in 2004, 2005, and 2006. For the UMD2 assembly, no sequences other than the BCM traces were used. We trimmed the reads to remove vector sequence using Figaro [10], which automatically determines vector sequence by identifying common prefixes in the reads. We trimmed the 3' end of the reads so that the mean error rate (computed from the quality scores) was <2.5% for any window of ≥ 40 bases. Our trimming and quality control routines yielded approximately 35 million trimmed reads, providing approximately 9.5× coverage of the genome. We next computed sequence overlaps among the trimmed reads using the UMD Overlapper [11,12], which includes an error-correction step that corrects sequencing errors in regions of sufficient coverage.

The sequencing strategy for *B. taurus *was a mixture of the WGS approach and a BAC-by-BAC approach. In the latter method, large-insert clones (BACs) of 100-150 Kbp are sequenced separately and then assembled. The WGS strategy, by contrast, samples the entire genome. For *B. taurus*, approximately 24 million reads were generated by WGS sequencing and approximately 11 million reads came from BACs. As a consequence, regions of the genome covered by BACs have significantly deeper coverage than the rest of the genome. This property in turn will confuse most WGS algorithms, which use coverage statistics to identify repetitive regions of a genome. To avoid this problem, we modified the Celera Assembler (CelAsm) program [13] to compute coverage and repeat statistics using only WGS reads. We then ran the modified CelAsm on the entire data set.

Further complicating the project was the fact that the source DNA originated from two animals, a father-daughter pair. The source of the BAC library DNA was Hereford bull L1 Domino 99375, registration number 41170496, with blood provided by Michael MacNeil's laboratory, USDA-ARS, Miles City, Montana. The DNA for the WGS sequences came from white blood cells from L1 Dominette 01449, American Hereford Association registration number 42190680 (a daughter of L1 Domino 99375), and was provided by Dr Timothy Smith's laboratory, US Meat Animal Research Center, Clay Center, Nebraska. The use of two animals increases the expected amount of diversity between haplotypes. Most of the reads were produced using a paired-end sequencing strategy, using clone inserts in two sets of sizes: several short libraries of 2-5 kb and several BAC-sized libraries of 150-200 kb.

Table S2 in Additional data file 1 summarizes the assembly after the initial run of CelAsm. The initial assembly contained 2.858 Gbp, with a maximum scaffold size of 15.1 Mbp and a total of 194,643 contigs. The initial contigs and scaffolds were mapped onto chromosomes and further improved as described below, and the final assembly statistics are shown in Table 1.

### Mapping the assembly onto the chromosomes

We used two sets of markers in the initial placement of the CelAsm scaffolds for UMD2: BAC ends from the IBBMC fingerprint map [4]; and the 17,524-marker composite map (Cmap) of Snelling and colleagues[4].

The fingerprint map (IBBMC) is a *Hin*DIII restriction map of 290,797 BACs that were assembled into 655 contigs and anchored on the *B. taurus *chromosomes [4]. Many of these BACs were end-sequenced from one or both ends, and we retrieved these sequences from the GSS database at NCBI. We were able to align 108,100 of BAC-end sequences onto our *B. taurus *genome assembly, using the requirement that each sequence align with >90% identity over >85% of its length. Most BAC ends matched with >98% of the sequence over >99% of their lengths. The MUMmer package [14] was used for these alignments and for the Cmap alignments. (The BCM4 marker positions for Cmap data were obtained directly from the BCM ftp site [15].) We manually examined some of the disagreements between FPmap and Cmap, and found that occasionally the FPmap appeared to jump to the wrong chromosome. Because Cmap is based on three independent sets of map data, we used Cmap to detect and correct such derailings and to create a 'corrected fingerprint map' (CFPmap). We then used this CFPmap to place our initial assembly onto the 30 *B. taurus *chromosomes. We also used CFPmap to correct 54 CelAsm scaffolds by splitting them into two or more pieces and separately placing the pieces onto chromosomes.

We then placed additional contigs and scaffolds onto the chromosomes if they were linked by three or more consistent mate-pair links to the placed scaffolds. We defined 'consistent' as: all mate pairs indicated the same relative orientation; and the standard deviation of the implied placement was consistent with that from the libraries for each mate pair.

### Orienting contigs using cow-human alignments

Scaffolds (sets of linked contigs) that were mapped onto chromosomes using only a single marker could not be oriented from the marker information alone. We oriented many of these scaffolds by taking advantage of the overall conserved synteny between cow and human. First, all cow scaffolds were aligned to the human genome using nucmer [14] with its maximal unique match (mum) option in order to avoid alignments of repetitive sequence. For each alignment of a previously unoriented scaffold to human, all alignments within 100 Kbp on each side were pulled out for analysis. A score *S *was computed for each unoriented scaffold, taking into account whether the scaffolds surrounding *S *on both sides (in cow) were mapped to a consistent set of locations in human. If the scaffolds surrounding *S *were oriented, and if a large majority of these scaffolds on both the left and right agreed on the orientation, then S was assigned that orientation. Using this procedure, 1,840 scaffolds containing 4,011 contigs were oriented.

We developed a similar procedure to assign unplaced contigs to chromosomes, again relying on conserved synteny between cow and human. First, all unplaced contigs were aligned as above. Mummer's 'delta-filter' program was then used to compute a one-to-one mapping of the unplaced contigs to human so that only the best aligning contig was considered at each region in human. For each unplaced contig's best alignment to human, the matching region in cow was identified via our human-cow syntenic map, and all contigs from this region were extracted for examination. We only considered placing a contig on a *B. taurus *chromosome if the order and direction of the surrounding contigs in cow matched the corresponding region in human. As above, we examined the alignments of nearby cow contigs that aligned within 100 kb of the unplaced contig's alignment in human. If the region of cow-human synteny contained no rearrangements, then the unplaced contig was placed at the location indicated by these alignments. Using this procedure, 1,046 contigs were placed on chromosomes. One consequence of this procedure was that a number of incompletely mapped genes (based on mRNA alignments) were completed.

### Haplotype variant removal

While evaluating the assembly for correctness, we found many examples of contigs placed along the chromosomes that aligned nearly identically with nearby contigs. When the two copies of each chromosome in a diploid genome diverge sufficiently, a genome assembler will be unable to merge the reads coming from the two haplotypes into a single consensus sequence. Instead, it partitions the reads into two separate contigs. In such cases, both contigs will have mate-pair links to surrounding contigs, and the assembler may place them very close to each other (usually adjacent) in the assembly. Although the ideal solution to this problem is to produce two complete copies of each chromosome, one for each parental haplotype, this solution is not possible with current technology. Therefore, we must retain one of the haplotypes and remove the other.

To detect and correct the haplotype variant problem, we aligned each contig to all contigs nearby. Those that aligned with >97% identity for >90% of their length were removed from the assembly and placed in a separate haplotype variants file. This procedure removed 3,010 contigs, totaling approximately 6 Mbp of sequence.

### Single nucleotide difference evaluation

We aligned the assemblies using the MUMmer suite of programs, and identified all positions where a 1-base mismatch was flanked by 50 bases that exactly matched on each side, and we further required that each assembly have at least 4 reads that aligned to these positions. Differences included substitutions, insertions and deletions. Note that this method excluded regions with multiple, closely spaced SNDs. We then matched all SND regions (101 bp each) against all *B. taurus *reads, seeding the alignments with exact 20-mer matches. An alignment of a SND to a read was considered valid if the entire SND region matched the assembly with a maximum of five errors.

For the comparison to the six finished *B. taurus *BACs, the following clones were downloaded from GenBank: gi|171461043, gi|171461042, gi|171461041, gi|171461040, gi|171461039, and gi|167744683. All six of these clones were sequenced and finished by BCM.

### Contig stitching

The scaffolder in CelAsm orders and orients the contigs into scaffolds based on the mate-pair relationships between reads. When the ends of contigs have low-quality, erroneous sequence, the scaffolder will place the contigs adjacent to one another and fail to merge them, even though the contigs actually overlap. To correct this problem, we post-processed scaffolds to replace overlapping contigs with a single joined contig, using a simplified version of the joining method described previously for the genome of *T. vaginalis *[16]. First, we aligned with nucmer [17] the ends of contigs estimated to have a gap between them of <1 Kbp. If the alignment showed that the contigs overlapped by at least 40 bp at 94% identity, with at most 20 bp of overhanging sequence, and the gap size implied by the overlap was <3 standard deviations of the estimated gap size, we stitched the pair of contigs together. The stitched sequence was composed of the left contig's sequence through the overlap region, concatenated with the region of the right flanking sequence past the overlap. The stitching processes each scaffold in order so chains of multiple contigs can be stitched together into a single large contig. The stitching process replaced 1,076 contigs (average size: 45.9 Kbp) with 534 joined contigs (average size: 91.7 Kbp), closing 542 gaps (average gap size: -822 bp).

### Gap closing by the 'shooting' method

Many of the gaps in a whole-genome assembly are due to repetitive sequences. For these sequences, an assembler must be very careful that it does not connect two non-contiguous regions of a genome. In many cases, gaps that remain after the assembler is finished can be resolved by carefully exploiting mate-pair information. We developed an algorithm to span gaps within a scaffold that enumerates all possible paths in the overlap graph (defined by overlapping reads). If exactly one of the paths is consistent with the mate-pair distances, then we can 'shoot' across the gap along that path. Using this algorithm, we were able to close 4,612 gaps, spanning approximately 8.34 Mbp in total.

### Human-cow syntenic map construction

The entire human genome was aligned to each chromosome of *B. taurus *using the MUMmer suite of programs, anchoring alignments with exact matches of at least 40 bp and requiring the alignment anchors to be at least 100 bp in length. Aligned regions ranged from 82 to 94% identity, and most alignments were 500-5,000 bp in length, likely corresponding to coding regions.

### Messenger RNA alignment

Known full-length gene sequences were downloaded from the RefSeq project at NCBI (release date: November 10, 2008) [18]. Of the 24,293 genes, only the 8,689 mRNAs promoted from experimentally validated sequences and identified with the code 'provisional' were retained. Sequences were aligned to the BCM4 and the UMD2 genomes using the high-throughput mapping tool ESTmapper [19,20], retaining all spliced alignments longer than 100 bp and with ≥ 95% sequence identity as significant. This procedure produced 12,069 alignments for 8,555 genes on the BCM4 genome and 12,460 alignments for 8,659 genes on the UMD2 genome, which were used to analyze the gene content of the two genomes. Alignments were also produced with an alternative mapping tool, GMAP [21], and used to confirm and classify the observed discrepancies in gene content between the two assemblies. For each gene, a 'coverage' value in each genome was computed as the fraction of its bases contained in all alignments of the gene, and the numbers of genes mapped at various coverage cutoffs were plotted.

For the human-cow gene alignments, we mapped 25,710 human proteins representing 18,109 unique gene IDs (in the NCBI RefSeq database) to the cow genome using tools that translated the genome in all six frames. The human genes were chosen by collecting all reviewed or validated RefSeq proteins that had explicit chromosomal coordinates. We performed cascading searches using blat, tblastn and exonerate to align the human proteins to DNA sequences, and we considered a protein present if it mapped across at least 40% of its length (with at least 70% similarity).

The complete assembly has been deposited at GenBank as accession DAAA00000000; the version described in this paper is the first version, DAAA01000000. The assembly is also on our ftp site [9].

## Abbreviations

BAC: bacterial artificial chromosome; BCM4: Baylor College of Medicine assembly of *B. taurus*, release 4; BtX: *B. taurus *X chromosome; CFPmap: corrected fingerprint map; HSB: homologous synteny block; HsX: *Homo sapiens *X chromosome; NCBI: National Center for Biotechnology Information; SND: single nucleotide difference; UMD2: University of Maryland assembly of *B. taurus*, release 2; WGS: whole-genome shotgun.

## Authors' contributions

AVZ, ALD, MCS, DP, and MR collected sequence data and ran assemblies. LF, FH, and GP aligned protein and transcript sequences and evaluated assembly completeness based on annotation. MCS, GM, MR, and PS re-assembled to close gaps and evaluated SNDs. CPVT and TSS provided mapping data and AVZ integrated map markers into the assembly. DAK and SLS aligned cow and human assemblies to improve orientation and to evaluate cow-human synteny. ALD and DP scanned for and removed contaminating sequences. JAY and SLS conceived the experiments and analyses. AVZ, ALD, LF, and SLS wrote the manuscript. All authors read and approved the final manuscript.

## Additional data files

The following additional data are available with the online version of this paper. Additional data file 1 contains two tables: Table S1 lists the number of RefSeq genes mapped to each of the two assemblies at varying levels of coverage; Table S2 lists the summary statistics for the initial, unimproved assembly of *B. taurus*. Additional data file 2 is a figure showing alignments between the UMD2 and BCM4 assemblies for all 30 chromosomes.

## Supplementary Material

Additional data file 1 Table S1 lists the number of RefSeq genes mapped to each of the two assemblies at varying levels of coverage; Table S2 lists the summary statistics for the initial, unimproved assembly of *B. taurus*. Click here for file

Additional data file 2 A PDF showing alignments between the UMD2 and BCM4 assemblies for all 30 chromosomes. Click here for file
