# Supplementary material for: A whole-genome assembly of the domestic cow, Bos taurus
Source: Genome Biol. 2009 Apr 24;10(4):R42. doi: 10.1186/gb-2009-10-4-r42 (PMC2688933; doi:10.1186/gb-2009-10-4-r42)
Supplement: Additional data file 1 — Table S1 lists the number of RefSeq genes mapped to each of the two assemblies at varying levels of coverage; Table S2 lists the summary statistics for the initial, unimproved assembly of B. taurus. [file gb-2009-10-4-r42-S1.doc]

**SUPPLEMENTARY DATA**

**Table S1.** Critical values for the number of RefSeq sequences (out of 8,689) that were mapped to each of the two genome assemblies when varying the coverage cutoff *x*. All alignments of a gene are considered. UMD2: the assembly created by the University of Maryland; BCM4: the assembly created by the Baylor College of Medicine.

| ***x*** | **BCM4** | **UMD2** |
| --- | --- | --- |
| 10 | 8,555 | 8,659 |
| 20 | 8,548 | 8,659 |
| 30 | 8,537 | 8,657 |
| 40 | 8,522 | 8,655 |
| 50 | 8,504 | 8,653 |
| 60 | 8,480 | 8,647 |
| 70 | 8,432 | 8,624 |
| 80 | 8,284 | 8,500 |
| 90 | 7,771 | 8,037 |
| 95 | 6,909 | 7,176 |
| 100 | 1,344 | 1,406 |

**Table S2**. Summary statistics for the initial, unimproved assembly of *Bos taurus* that formed the basis of the UMD2 genome. N50 scaffold (contig) size is defined as the size *k* such that 50% of the genome is contained in scaffolds (contigs) larger than *k*. The coverage of the entire genome based on this assembly is 8.2-fold; i.e., each base in each contig is contained in an average of 8.2 reads.

| Total number of scaffolds | 134,612 |
| --- | --- |
| Total number of contigs | 194,643 |
| Total size of all contigs (bp) | 2,858,277,365 |
| Maximum scaffold size (bp) | 15,072,136 |
| N50 scaffold size (bp), based on genome size of 2.86 Gbp | 2,055,041 |
| Maximum contig size (bp) | 742,802 |
| N50 contig size (bp), based on genome size of 2.86 Gbp | 74,604 |
| Number of contigs > 10,000 bp | 45,939 |
| Total size of contigs > 10,000 bp | 2,546,781,351 |
