# Supplementary figures and images for: A whole-genome assembly of the domestic cow, Bos taurus
Source: Genome Biol. 2009 Apr 24;10(4):R42. doi: 10.1186/gb-2009-10-4-r42 (PMC2688933; doi:10.1186/gb-2009-10-4-r42)

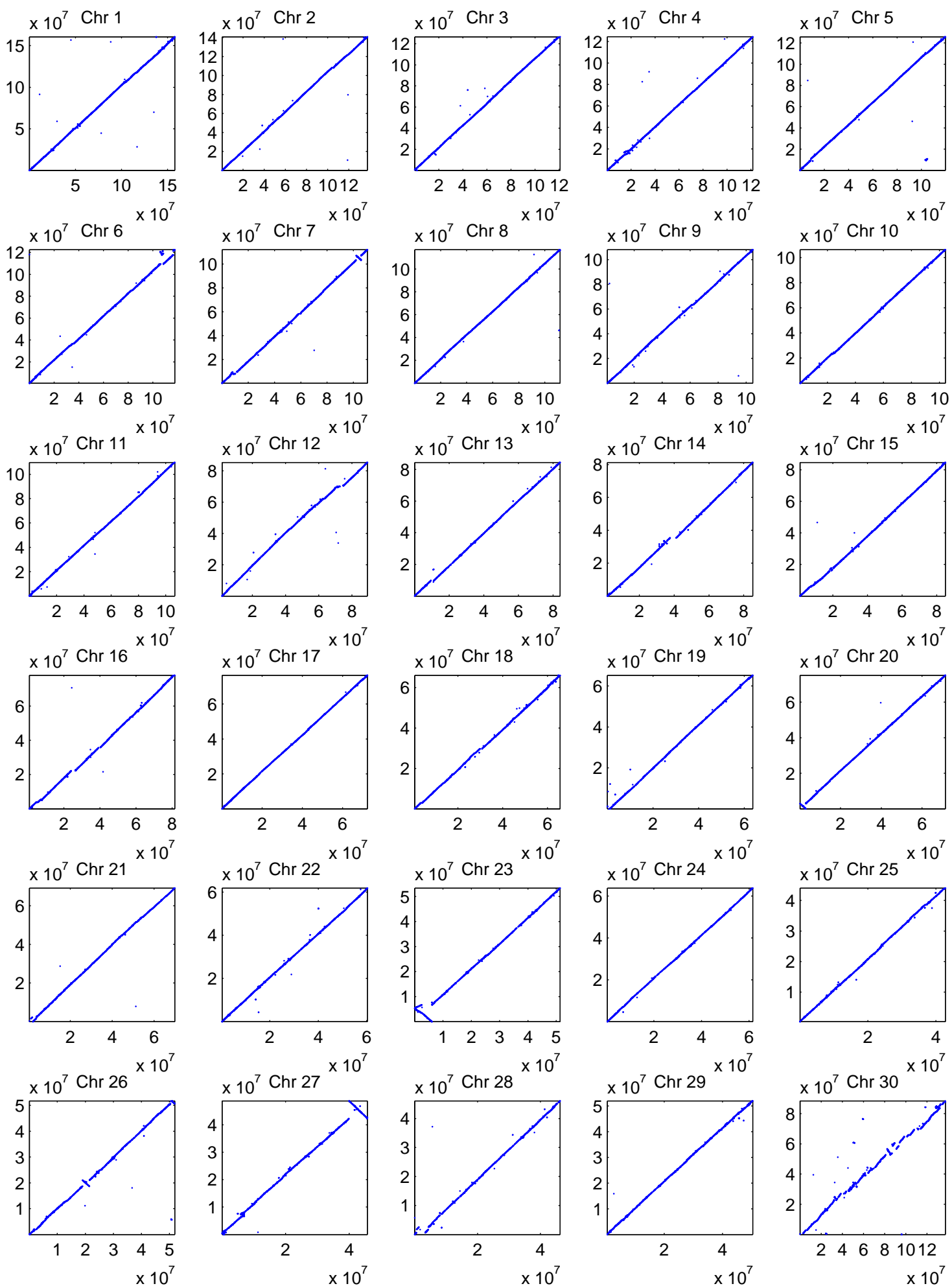

Supplement: Additional data file 2 — A PDF showing alignments between the UMD2 and BCM4 assemblies for all 30 chromosomes. [file gb-2009-10-4-r42-S2.pdf]
